# 

**Published:** 1914-03

**Authors:** 


### IMPORTANT MEETING

The Medical Association of Georgia will meet in Atlanta, April 15, 16 and 17. A large attendance is expected and an interesting, scientific program has been arranged. A number of social intertainments will be provided and it is sincerely hoped that the physicians of Georgia will avail themselves of Atlanta's hospitality on this occasion. No one hotel could furnish the desired accomodations, so there will be no general headquarters except at the Wesley Memmorial Church on Auburn Avenue, where the meetings will be held. Full details of the scientific and social features will be supplied at the regrestration booth. Atlanta extends a most cordial invitation to the physicians of Georgia.

